# Transcriptional Profiles of Host-Pathogen Responses to Necrotic Enteritis and Differential Regulation of Immune Genes in Two Inbreed Chicken Lines Showing Disparate Disease Susceptibility

**DOI:** 10.1371/journal.pone.0114960

**Published:** 2014-12-11

**Authors:** Duk Kyung Kim, Hyun S. Lillehoj, Seung I. Jang, Sung Hyen Lee, Yeong Ho Hong, Hans H. Cheng

**Affiliations:** 1 Animal Biosciences and Biotechnology Laboratory, Agricultural Research Service, U.S. Department of Agriculture, Beltsville, MD, 20705, United States of America; 2 Animal Genomics and Breeding Center, Hankyong National University, Anseong, Gyeonggi, 456–749, South Korea; 3 National Academy of Agricultural Science, Rural Development Administration, Wanju-Gun, Jeollabuk-do, 565–851, South Korea; 4 Department of Animal Science and Technology, Chung-Ang University, Anseong, 456–756, South Korea; 5 Avian Disease and Oncology Laboratory, Agricultural Research Service, U.S. Department of Agriculture, East Lansing, MI, 48823, United States of America; University of New England, Australia

## Abstract

Necrotic enteritis (NE) is an important intestinal infectious disease of commercial poultry flocks caused by *Clostridium perfringens*. Using an experimental model of NE involving co-infection with *C. perfringens* and *Eimeria maxima*, transcriptome profiling and functional genomics approaches were applied to identify the genetic mechanisms that might regulate the host response to this disease. Microarray hybridization identified 1,049 transcripts whose levels were altered (601 increased, 448 decreased) in intestinal lymphocytes from *C. perfringens*/*E. maxima* co-infected Ross chickens compared with uninfected controls. Five biological functions, all related to host immunity and inflammation, and 11 pathways were identified from this dataset. To further elucidate the role of host genetics in NE susceptibility, two inbred chicken lines, ADOL line 6 and line 7 which share an identical *B^2^* major histocompatibility complex haplotype but differ in their susceptibility to virus infection, were compared for clinical symptoms and the expression levels of a panel of immune-related genes during experimental NE. Line 6 chickens were more susceptible to development of experimental NE compared with line 7, as revealed by decreased body weight gain and increased *E. maxima* oocyst shedding. Of 21 immune-related genes examined, 15 were increased in *C. perfringens*/*E. maxima* co-infected line 6 *vs*. line 7 chickens. These results suggest that immune pathways are activated in response to experimental NE infection and that genetic determinants outside of the chicken *B* complex influence resistance to this disease.

## Introduction

Necrotic enteritis (NE) is an acute clostridial disease of economic importance to the poultry industry [1]. NE is caused by *Clostridium perfringens*, a Gram-positive, rod-shaped, spore-forming, and oxygen-tolerant anaerobe [2]. *C. perfringens* is a normal component of the chicken gut microbiota and the alterations in the host-pathogen relationship that regulate the development of NE remain to be determined [3]. A variety of predisposing factors, however, are known to promote disease, including diets containing high levels of wheat, barley, or poorly digestible proteins and co-infection by the apicomplexan protozoa, *Eimeria*, the etiologic agent of avian coccidiosis [4], [5]. Because of the risk of transmission to humans through the food chain, *C. perfringens* also is important for public health [4], [6]. Traditionally, prophylactic in-feed antibiotics have proven effective for control of clostridial field infections in chickens. However, NE has recently emerged as a significant problem as a result of decreased antibiotic usage amid concerns over the appearance of antibiotic-resistant human pathogens [4], [7].

Only limited information exists on the role of genetic factors in controlling the susceptibility of chickens to NE. Since many common host immune responses are elicited following infection by viral, bacterial, or parasitic pathogens [9], we hypothesized that two inbred chicken lines with dissimilar susceptibility to virus-induced diseases might provide insights into the genetic mechanisms regulating avian NE. White Leghorn USDA Avian Disease and Oncology Laboratory (ADOL) line 6 and line 7 chickens are 99% inbred and possess the major histocompatibility complex (MHC) *B^2^* genotype, but differ in their immunoglobulin G allotype (*G1^E^* and *G1^A^*, respectively). Both lines were originally selected based on their susceptibility to avian leukosis retroviruses (ALV) and Marek's diseases herpesviruses (MDV), with line 6 being the more susceptible to both viruses [8]. Although susceptibility to Marek's disease has not been associated with co-susceptibility *Eimeria* infection [9], line 6 chickens do exhibit greater susceptibility to *E. maxima* infection compared with line 7 [10]. However, no published studies have examined NE susceptibility in these two chicken lines. Therefore, the objectives of the current study were (1) to determine whether ADOL line 6 and line 7 exhibit different susceptibilities to experimental NE using a *C. perfringens*/*E. maxima* co-infection model and (2) to compare transcriptional profiles in co-infected *vs*. uninfected Ross chickens in order to identify candidate genes that might be differentially expressed in NE-afflicted line 6 *vs*. line 7 birds.

## Materials and Methods

### Experimental animals

Commercial Ross x Ross broilers were obtained from Longenecker's Hatchery (Elizabethtown, PA). ADOL line 6 and line 7 chickens were hatched at the USDA Avian Disease and Oncology Laboratory (East Lansing, MI). All chickens were housed in Petersime starter brooder units in an *Eimeria*-free facility for 14 days post-hatch and provided with feed and water *ad libitum*. Chickens were transferred to larger hanging cages with 2 birds/cage at a separate location where they were co-infected with *C. perfringens* and *E. maxima* and kept until the end of the experimental period.

### Experimental NE disease model

Chickens were infected with *E. maxima* strain 41A (1.0×10^4^ oocysts/bird) by oral gavage on day 14 post-hatch followed by infection with *C. perfringens* strain Del-1 (1.0×10^9^ colony forming units/bird) by oral gavage on day 18 [11]. To facilitate development of NE, birds were fed an antibiotic-free, certified organic starter diet containing 17% crude protein between days 1–18 followed by a standard grower diet containing 24% crude protein between days 18–20. Uninfected control animals were housed in neighboring cages and given the same diet. Body weight gains (16 birds/group) were measured and fecal samples were collected between days 14–20 post-hatch (days 0–9 post-infection with *E. maxima*). Oocyst numbers were determined using a McMaster chamber (HK Inc., Tokyo, Japan) [12]. Gut lesion scores were determined at day 20 post-hatch (day 2 post-infection with *C. perfringens*) on a scale from 0 (none) to 4 (high) in a blinded fashion by three independent observers as described [13], [14]. All protocols were approved by the USDA Beltsville Area Institutional Animal Care and Use Committee.

### RNA extraction from intestinal intraepithelial lymphocytes (IELs)

Total RNA was isolated from intestinal IELs of Ross chickens as described [15]. Briefly, the intestinal jejunum was removed at day 20 post-hatch, cut longitudinally, and washed three times with ice-cold Hank's balanced salt solution (HBSS). Harvested tissues were incubated in HBSS containing 0.5 mM EDTA and 5% FBS for 20 min at 37°C with constant swirling. Cells released into the supernatant were passed through nylon wool (Robbins Scientific, Sunnyvale, CA), and washed twice with HBSS containing 5% FBS. IELs were purified by Percoll density gradient centrifugation and washed three times with HBSS containing 5% FBS. Total RNA was isolated using Trizol (Invitrogen, Grand Island, NY).

### Microarray hybridization

RNA from 6 birds was pooled into 2 equal weight samples including RNA from 3 birds each. Each sample was labeled with cyanine 3 (Cy3)- or Cy5-CTP using the Two-Color Quick Amp Labeling Kit (Agilent Technologies, Santa Clara, CA) as described [16]. Labeled RNAs from uninfected and *C. perfringens*/*E. maxima* co-infected chickens were hybridized with the Chicken Gene Expression Microarray (Agilent Technologies) containing 43,803 elements. Four hybridizations were conducted with alternation of Cy3- and Cy5-labeled RNAs to prevent data distortion from sample labeling [17]. Microarray images were scanned and data extraction and analysis were performed using Feature Extraction software version 10.7.3.1 (Agilent Technologies).

### Microarray data analysis

GeneSpring GX10 software (Agilent Technologies) was used to qualify and normalize image analysis data and to determine the fold changes in gene expression. Average signal intensities were corrected for background signals and normalized by block locally-weighted regression scatterplot smoothing (LOWESS). Flag information, the feature quality data from the signals on microarray, was applied to the spots without blank or invalid values from each sample asymptotic t-test analysis with *P*<0.05 was performed to analyze the significance of the differences between the uninfected and NE-afflicted groups. To generate signal ratios, signal channel values from NE-afflicted birds were divided by values from uninfected negative controls. All microarray information and data were deposited in the Gene Expression Omnibus database (series record number, GSE51154). Differentially expressed genes between NE-afflicted and uninfected control chickens were analyzed using Ingenuity Pathway Analysis (IPA) software (Ingenuity Systems, Redwood City, CA). Each identifier was mapped to its corresponding gene in IPA. These identified genes were superimposed onto the global molecular networks contained within IPA. IPA functional analysis was performed to identify the biological functions associated with the identified genes from the mapped datasets.

### Quantitative (q)RT-PCR

Equivalent amounts of the same intestinal IEL RNA used for microarray hybridization from uninfected or *C. perfringens*/*E. maxima* co-infected Ross chickens, and fresh IEL RNA from uninfected or co-infected ADOL line 6 and line 7 chickens, were reverse-transcribed using the AffinityScript Multiple Temperature cDNA Synthesis Kit (Agilent Technologies) as described [20]. PCR amplification was performed using the oligonucleotide primers listed in Table 1 with the Mx3000P system and Brilliant SYBR Green qRT-PCR master mix (Agilent Technologies). Standard curves were generated using log_10_ diluted standard RNAs and the levels of individual transcripts were normalized to those of glyceraldehyde-3-phosphate dehydrogenase (GAPDH) by the Q-gene program [18]. The cycle threshold (C_t_) value of the target gene was normalized to GAPDH and calibrated to the relevant control value. Each analysis was performed in triplicate.

**Table 1:** Oligonucleotide primers used for qRT-PCR.

Gene Symbol		Primer Sequence (5′→3′)	Entrez Gene Name	GenBank Accession No.
LCP1	Forward	GCCAGTAGACTGGAACAGAG	Lymphocyte cytosolic protein 1 (L-plastin)	NM_001008440.1
	Reverse	TTCTCTCCACCACCAATATC		
MTTP	Forward	TTCCTATATGCCTGTGGATT	Microsomal triglyceride transfer protein	NM_001109784.1
	Reverse	AGGTACATCCTCACGTTGTC		
CALB1	Forward	GCTTGGACTTAACACCTGAA	Calbindin 1, 28 kDa	NM_205513.1
	Reverse	TCCTCAGAATCAATGAAACC		
CXCL14	Forward	CCAGTGCAGAAGGAGTAAAG	Chemokine (C-X-C motif) ligand 14	NM_204712.2
	Reverse	TTCCATACTCGGTACCACTT		
ANXA1	Forward	CAATGATGCAAGGGCCTTAT	Annexin A1	NM_206906.1
	Reverse	CTTCATTGCCAGGTGGAGTT		
IL8	Forward	GGCTTGCTAGGGGAAATGA	Interleukin 8	AJ009800
	Reverse	AGCTGACTCTGACTAGGAAACTGT		
APP	Forward	GGAAGCGATGATAAGGTGGTAGAAGAACAA	Amyloid beta (A4) precursor protein	NM_204308
	Reverse	CATCACCATCATCATCGTCATCATCATCAG		
ARHGEF6	Forward	ACTGCTGGGAAATGTGGAGGAAATC	Rac/Cdc42 guanine nucleotide exchange factor (GEF) 6	NM_001006432
	Reverse	ACGTCAGGTACAGGGAGCGGAACT		
BCL2	Forward	GATGTGCGTCGAGAGCGTCAA	B-cell CLL/lymphoma 2	NM_205339
	Reverse	GTGCAGGTGCCGGTTCAGGT		
COL1A2	Forward	CTCAGCTTTGTGGATACGCGGATTTTG	Collagen, type I, alpha 2	NM_001079714
	Reverse	GCCCTGCAGATGCCTCACTCACA		
CXCL14	Forward	GCCTTGCTTCTGCTGGTCATC	Cchemokine (C-X-C motif) ligand 14	NM_204712
	Reverse	ATCTTATTTTCGGCCCTTTCCTT		
GJA1	Forward	GTCTTCATGCTGGTAGTGTCTTTGGTGTCT	Gap junction protein, alpha 1, 43 kDa	NM_204586
	Reverse	CTGTGGGAGTAGGGGTCGGTTTTTC		
HSP90B1	Forward	CTGGCTCTGGCATGCACGCTTCT	Heat shock protein 90 kDa beta (Grp94), member 1	NM_204289
	Reverse	CTTCATCATCAGTTCGGGACCCTTCTCTAC		
NFKBIZ	Forward	CCAGGTCCTCCAGGCAATCCAAAAG	Nuclear factor of kappa light polypeptide gene enhancer in B-cells inhibitor, zeta	NM_001006254
	Reverse	AGTGCAGGGCTGTCAAACCATCGTAG		
SERPINF1	Forward	CGGCAGCAGACAAGGGGAAGGATT	Serpin peptidase inhibitor, clade F, member 1	NM_001257289
	Reverse	TGAAGTAAGCAGCCCCAGCAAGGAG		
SOCS3	Forward	GACACCAGCCTGCGCCTCAAGA	Suppressor of cytokine signaling 3	NM_204600
	Reverse	GCCCGTCACCGTGCTCCAGTAGA		
SOCS6	Forward	CAGATATCTTTGTGGACCAGGCAGTGAA	Suppressor of cytokine signaling 6	NM_001127312
	Reverse	GGTAGCAAAGGTGAAAGTGGAGGGACATC		
TAB3	Forward	CACCGCAAAGACCTGGGACTG	TGF-beta activated kinase 1/MAP3K7 binding protein 3	XM_416787
	Reverse	GTGGGTGCTGGTTTCGTTGAGATGGT		
TCF12	Forward	CTCGGGGAAACCTGGAACAACCTACTA	Transcription factor 12	NM_205375
	Reverse	GGGGGCACCTTTCTTACTTTCTTTGTCT		
TNFRSF11B	Forward	CATCTCGGCCAACCAAGTCTCACCT	Tumor necrosis factor receptor superfamily, member 11 b	NM_001033641
	Reverse	CGCTCGATATCTTCTTTTCCCACTTTCTTG		
TRAF3	Forward	CGTCTCGGCGCCACTTAGGA	TNF receptor-associated factor 3	XM_421378
	Reverse	GGGCAGCCAGACGCAATGTTCA		
VEGFA	Forward	GGCCTAGAATGTGTCCCTGTGG	Vascular endothelial growth factor A	NM_001110355
	Reverse	ATGTGCGCTATGTGCTGACTCTGA		
LITAF	Forward	TGTGTATGTGCAGCAACCCGTAGT	Lipopolysaccharide-induced TNF factor	AY765397
	Reverse	GGCATTGCAATTTGGACAGAAGT		
GAPDH	Forward	TGCTGCCCAGAACATCATCC	Glyceraldehyde-3-phosphate dehydrogenase	NM_204305
	Reverse	ACGGCAGGTCAGGTCAACAA		

### Statistical analyses

Data from body weight gains, lesion scores, oocyst shedding, and mRNA expression levels were expressed as the mean ± SD values. Comparisons of the mean values were performed by ANOVA and the Student's t-test using SPSS software (SPSS 15.0 for Windows, Chicago, IL) and were considered significant at *P*<0.05. IPA biological function analysis was performed using the Fisher's exact test to calculate the probability of each biological function assigned to that dataset and were considered significant at *P*<0.05.

## Results

### Effects of experimental NE on body weight gain and lesion score

**Figure 1:**
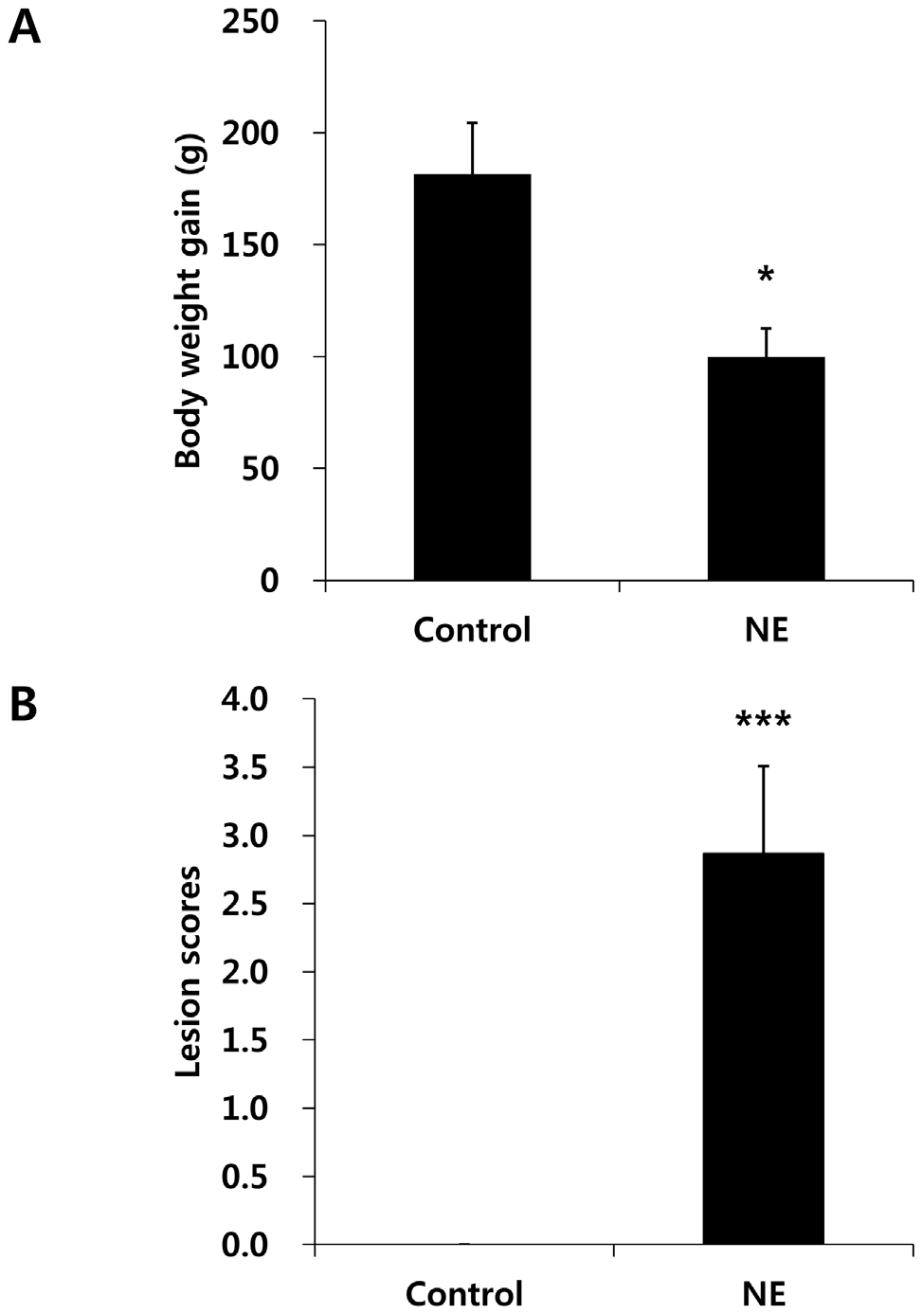
Effect of *C. perfringens*/*E. maxima* co-infection of Ross chickens on body weight gains and intestinal lesion scores. (A) Chickens were uninfected (Control) or co-infected with *C. perfringens* and *E. maxima* (NE) and body weight gains were measured between days 0–9 post-infection with *E. maxima*. (B) Gut lesion scores were determined at day 2 post-infection with *C. perfringens* on a scale from 0 (none) to 4 (high) in a blinded fashion by three independent observers. Each bar represents the mean ± SD value (n = 8). *, *P*<0.05; ***, *P*<0.001.

Co-infection of Ross chickens with *C. perfringens* and *E. maxima* reduced body weight gain between days 0–9 post-infection with *E. maxima* by 45% compared with uninfected birds (uninfected gain, 182±22 g; co-infected gain, 100±12 g) (Fig. 1A). Co-infected chickens also had higher gut lesion scores at day 2 post-infection with *C. perfringens* compared with uninfected chickens (uninfected score 0±0; co-infected score, 2.9±0.6) (Fig. 1B).

### Effects of NE on global gene expression in intestinal IELs

Microarray hybridization analysis identified 1,049 transcripts in intestinal IELs whose levels were significantly altered beyond a cutoff of>2.0-fold at day 2 post-infection with *C. perfringens* in *C. perfringens*/*E. maxima* co-infected Ross chickens compared with uninfected controls. Of these, 601 transcript levels were increased and 448 were decreased. This dataset was mapped to the human, mouse, rat, and chicken genomes using Ingenuity Knowledge Base software, leading to the identification and annotation of 462 chicken genes. The most 20 most up-regulated and 20 most down-regulated transcripts in co-infected birds compared with uninfected controls are listed in Table 2.

**Table 2:** The 20 most up-regulated and 20 most down-regulated intestinal IEL transcript levels in *C. perfringens*/*E. maxima* co-infected Ross chickens compared with uninfected controls.

Symbol	Entrez Gene Name	Fold Change	Location	Type(s)
**Up-regulated**				
MMP7	Matrix metallopeptidase-7	45.476	Extracellular Space	Peptidase
LYG2	Lysozyme G-like 2	35.509	Extracellular Space	Enzyme
VCAM1	Vascular cell adhesion molecule 1	14.508	Plasma Membrane	Other
ATP13A4	ATPase type 13A4	12.263	Unknown	Transporter
BATF3	Basic leucine zipper transcription factor, ATF-like 3	11.368	Nucleus	Transcription Regulator
TAC1	Tachykinin, precursor 1	9.837	Extracellular Space	Other
C3	Complement component 3	9.8	Extracellular Space	Peptidase
SOCS1	Suppressor of cytokine signaling 1	9.571	Cytoplasm	Other
LAG3	Lymphocyte-activation gene 3	9.217	Plasma Membrane	Transmembrane Receptor
GLDC	Glycine dehydrogenase (decarboxylating)	8.991	Cytoplasm	Enzyme
IL21R	Interleukin 21 receptor	8.101	Plasma Membrane	Transmembrane Receptor
RRM2	Ribonucleotide reductase M2	7.797	Nucleus	Enzyme
SAMHD1	SAM domain and HD domain 1	7.729	Nucleus	Enzyme
MIS12	MIS12, MIND kinetochore complex component, homolog (S. pombe)	7.474	Nucleus	Other
CSF2RA	Colony stimulating factor 2 receptor, alpha, low-affinity (granulocyte-macrophage)	7.444	Plasma Membrane	Transmembrane Receptor
IRG1	Immunoresponsive 1 homolog (mouse)	7.171	Unknown	Other
MDFI	MyoD family inhibitor	7.161	Cytoplasm	Other
TRAIP	TRAF interacting protein	7.09	Cytoplasm	Other
SFTPA1	Surfactant protein A1	7.003	Extracellular Space	Transporter
SMC2	Structural maintenance of chromosomes 2	6.872	Nucleus	Transporter
**Down-regulated**				
C13orf15	Chromosome 13 open reading frame 15	−104.868	Cytoplasm	Other
AGXT2L1	Alanine-glyoxylate aminotransferase 2-like 1	−70.677	Unknown	Enzyme
CMBL	Carboxymethylenebutenolidase homolog (Pseudomonas)	−28.026	Unknown	Enzyme
HES6	Hairy and enhancer of split 6 (Drosophila)	−25.238	Nucleus	Transcription Regulator
RBPMS2	RNA binding protein with multiple splicing 2	−22.282	Unknown	Other
S100B	S100 calcium binding protein B	−17.322	Cytoplasm	Other
SERPINF1	Serpin peptidase inhibitor, clade F (alpha-2 antiplasmin, pigment epithelium derived factor), member 1	−17.216	Extracellular Space	Other
SERPINB2	Serpin peptidase inhibitor, clade B (ovalbumin), member 2	−16.241	Extracellular Space	Other
BBOX1	Butyrobetaine (gamma), 2-oxoglutarate dioxygenase (gamma-butyrobetaine hydroxylase) 1	−15.873	Cytoplasm	Enzyme
RHAG	Rh-associated glycoprotein	−13.518	Plasma Membrane	Peptidase
CXCL14	Chemokine (C-X-C motif) ligand 14	−13.029	Extracellular Space	Cytokine
MYL10	Myosin, light chain 10, regulatory	−12.77	Cytoplasm	Other
TMEM199	Transmembrane protein 199	−12.112	Unknown	Other
ANXA1	Annexin A1	−11.609	Plasma Membrane	Other
NRG4	Neuregulin 4	−11.399	Extracellular Space	Growth Factor
ALDH1A1	Aldehyde dehydrogenase 1 family, member A1	−10.85	Cytoplasm	Enzyme
RNF152	Ring finger protein 152	−10.558	Cytoplasm	Enzyme
COL1A2	Collagen, type I, alpha 2	−10.196	Extracellular Space	Other
SYNM	Synemin, intermediate filament protein	−9.059	Cytoplasm	Other
CALB1	Calbindin 1, 28 kDa	−8.966	Cytoplasm	Other

### Effects of NE on biological function, pathway, and network analysis of differentially regulated IELs transcripts

Five significantly altered biological functions were identified from the differently altered genes in intestinal IELs from *C. perfringens*/*E. maxima* co-infected Ross chickens compared with uninfected controls (Table 3). The *P* value associated with a particular function in this analysis is a statistical measure of the likelihood that genes from the dataset under investigation participate in that function. All of the 5 identified biological functions were related to the immune response, particularly the quantity and movement of leukocytes. The most significant function annotated was “immune response” including a category of “inflammatory response” with 83 genes, followed by “cell movement of leukocytes” including the categories of “cellular movement,” “hematological system development and function,” and “immune cell trafficking” with 49 genes.

**Table 3:** The biological functions significantly associated with the differentially expressed transcripts in *C. perfringens*/*E. maxima* co-infected Ross chickens compared with uninfected controls.

Functions Annotation	Category	*P* Value	No. of Genes
Immune response	Inflammatory Response	5.93E-12	83
Cell movement of leukocytes	Cellular Movement	1.36E-09	49
	Hematological System Development and Function		
	Immune Cell Trafficking		
Quantity of leukocytes	Hematological System Development and Function	1.96E-09	45
	Tissue Morphology		
Cell movement of blood cells	Cellular Movement	2.21E-09	53
Quantity of mononuclear leukocytes	Hematological System Development and Function	2.52E-09	38
	Tissue Morphology		

Canonical pathway analysis of the differentially expressed genes in co-infected *vs*. uninfected chickens identified 11 pathways (Table 4). The most significant of these were “leukocyte extravasation signaling,” “relaxin signaling,” and “LPS/IL-1 mediated inhibition of RXR function”, which were associated with 14, 11, and 13 genes, respectively (Table 5).

**Table 4:** The IPA pathways significantly associated with the differentially expressed transcripts in *C. perfringens*/*E. maxima* co-infected Ross chickens compared with uninfected controls.

Ingenuity Canonical Pathway	*P* Value	Ratio*
Leukocyte Extravasation Signaling	1.58E-03	7.00E-02
Relaxin Signaling	1.82E-03	6.96E-02
LPS/IL-1 Mediated Inhibition of RXR Function	2.29E-03	5.83E-02
Pyruvate Metabolism	2.82E-03	5.19E-02
Neuroprotective Role of THOP1 in Alzheimer's Disease	2.88E-03	9.26E-02
HIF1α Signaling	2.95E-03	8.33E-02
ATM Signaling	7.08E-03	1.11E-01
HMGB1 Signaling	7.41E-03	8.00E-02
Tight Junction Signaling	8.32E-03	6.10E-02
ILK Signaling	9.33E-03	6.22E-02
Hepatic Fibrosis/Hepatic Stellate Cell Activation	9.77E-03	6.80E-02

^*^The ratio of the number of genes from the dataset mapped to the indicated pathway divided by the total number of genes within that particular pathway.

**Table 5:** Genes included in the three canonical pathways most significantly associated with the differentially expressed transcripts in *C. perfringens*/*E. maxima* co-infected Ross chickens compared with uninfected controls.

Pathway	Gene Symbol	Entrez Gene Name	Fold Change	Location	Type(s)
Leukocyte Extravasation Signaling	CD99	CD99 molecule	−4.524	Plasma Membrane	Other
	ACTC1	Actin, alpha, cardiac muscle 1	−3.201	Cytoplasm	Enzyme
	CTTN	Cortactin	−2.679	Plasma Membrane	Other
	MAPK9	Mitogen-activated protein kinase 9	−2.154	Cytoplasm	Kinase
	ARHGAP12	Rho GTPase activating protein 12	−2.1	Cytoplasm	Other
	CLDN3	Claudin 3	−2.098	Plasma Membrane	Transmembrane Receptor
	FER	Fer (fps/fes related) tyrosine kinase	2.279	Cytoplasm	Kinase
	PIK3R5	Phosphoinositide-3-kinase, regulatory subunit 5	2.584	Cytoplasm	Kinase
	MMP9	Matrix metallopeptidase 9 (gelatinase B, 92kDa gelatinase, 92kDa type IV collagenase)	3.071	Extracellular Space	Peptidase
	NCF2	Neutrophil cytosolic factor 2	3.464	Cytoplasm	Enzyme
	PIK3CD	Phosphoinositide-3-kinase, catalytic, delta polypeptide	3.775	Cytoplasm	Kinase
	RAC2	Ras-related C3 botulinum toxin substrate 2 (rho family, small GTP binding protein Rac2)	3.919	Cytoplasm	Enzyme
	VCAM1	Vascular cell adhesion molecule 1	14.508	Plasma Membrane	Other
	MMP7	Matrix metallopeptidase 7 (matrilysin, uterine)	45.476	Extracellular Space	Peptidase
Relaxin Signaling	MPPE1	Metallophosphoesterase 1	2.005	Cytoplasm	Enzyme
	PDE3B	Phosphodiesterase 3B, cGMP-inhibited	2.18	Cytoplasm	Enzyme
	PIK3CD	Phosphoinositide-3-kinase, catalytic, delta polypeptide	3.775	Cytoplasm	Kinase
	PIK3R5	Phosphoinositide-3-kinase, regulatory subunit 5	2.584	Cytoplasm	Kinase
	MMP9	Matrix metallopeptidase 9 (gelatinase B, 92kDa gelatinase, 92kDa type IV collagenase)	3.071	Extracellular Space	Peptidase
	VEGFA	Vascular endothelial growth factor A	−2.245	Extracellular Space	Growth Factor
	CREB1	cAMP responsive element binding protein 1	2.05	Nucleus	Transcription Regulator
	ADCY5	Adenylate cyclase 5	−2.53	Plasma Membrane	Enzyme
	ADCY9	Adenylate cyclase 9	−3.136	Plasma Membrane	Enzyme
	GNA11	Guanine nucleotide binding protein (G protein), alpha 11 (Gq class)	−2.271	Plasma Membrane	Enzyme
	GNG13	Guanine nucleotide binding protein (G protein), gamma 13	−3.434	Plasma Membrane	Enzyme
LPS/IL-1 Mediated Inhibition of RXR Function	ACSBG2	Acyl-CoA synthetase bubblegum family member 2	2.046	Cytoplasm	Enzyme
	ACSL5	Acyl-CoA synthetase long-chain family member 5	4.067	Cytoplasm	Enzyme
	ALDH1A1	Aldehyde dehydrogenase 1 family, member A1	−10.85	Cytoplasm	Enzyme
	CYP3A4	Cytochrome P450, family 3, subfamily A, polypeptide 4	2.265	Cytoplasm	Enzyme
	FABP5	Fatty acid binding protein 5 (psoriasis-associated)	−2.642	Cytoplasm	Transporter
	GSTT1	Glutathione S-transferase theta 1	−2.622	Cytoplasm	Enzyme
	HMGCS2	3-hydroxy-3-methylglutaryl-CoA synthase 2 (mitochondrial)	−6.489	Cytoplasm	Enzyme
	MAPK9	Mitogen-activated protein kinase 9	−2.154	Cytoplasm	Kinase
	SMOX	Spermine oxidase	−2.372	Cytoplasm	Enzyme
	HS6ST1	Heparan sulfate 6-O-sulfotransferase 1	2.632	Plasma Membrane	Enzyme
	SLC27A4	Solute carrier family 27 (fatty acid transporter), member 4	2.839	Plasma Membrane	Transporter
	TNFRSF11B	Tumor necrosis factor receptor superfamily, member 11b	−4.19	Plasma Membrane	Transmembrane Receptor
	TNFRSF1B	Tumor necrosis factor receptor superfamily, member 1B	4.62	Plasma Membrane	Transmembrane Receptor

**Figure 2:**
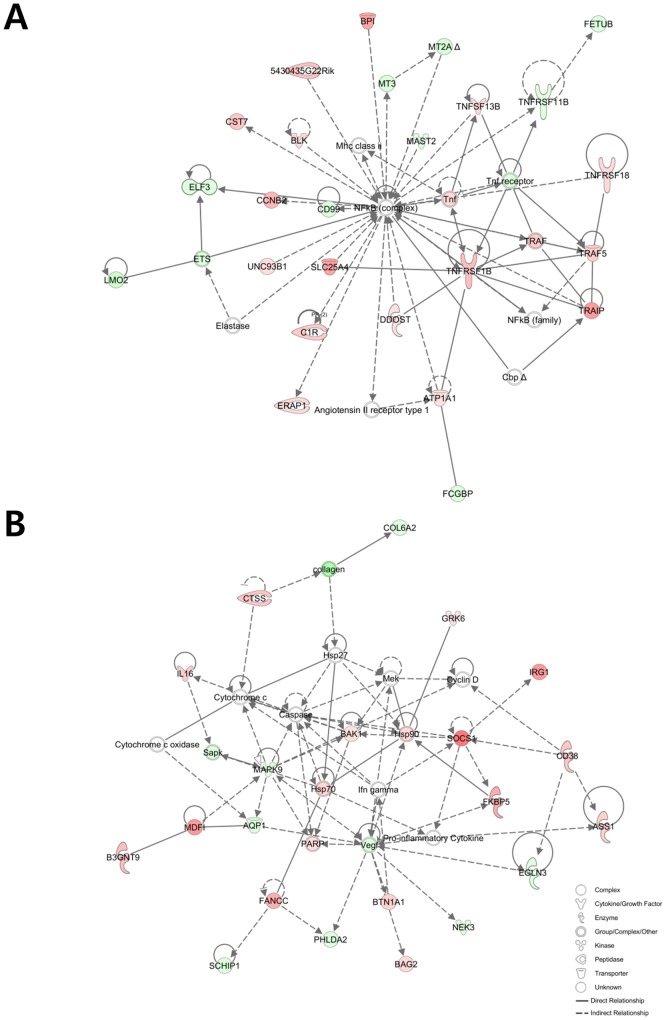
The first (A) and seventh (B) networks of genes most significantly associated with the differentially expressed transcripts in *C. perfringens*/*E. maxima* co-infected Ross chickens compared with uninfected controls. Up- and down-regulated genes are illustrated by red and green, respectively. The color intensity directly correlates with the difference in the expression level of the corresponding gene compared between line 6 and line 7 chickens.

Twenty-five networks related to the differentially expressed genes in co-infected *vs*. uninfected chickens were identified by IPA network analysis (S1 Table). Two of these networks were related to the immune response, including the most significantly associated network containing 25 focused genes and associated with the biological functions “cellular function and maintenance”, “inflammatory response”, and “cell-to-cell signaling and interaction” (Fig. 2A). The seventh most significantly associated network also was related to the immune response and contained 21 focused genes associated with the biological functions “cell death,” “inflammatory response,” and “organismal injury and abnormalities” (Fig. 2B).

### Validation of microarray analysis by qRT-PCR

**Figure 3:**
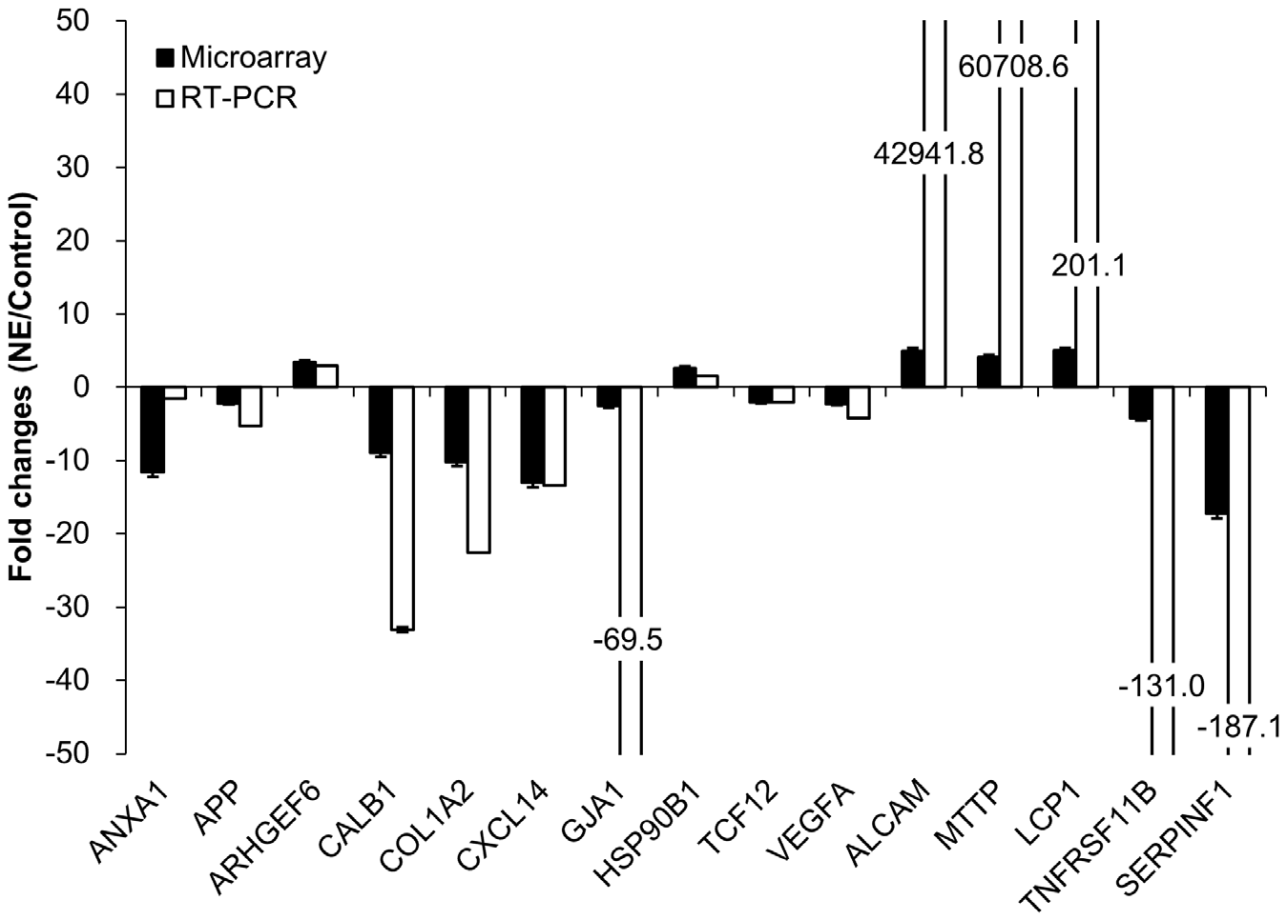
Comparison of the fold changes in the expression levels of 15 immune-related gene transcripts in intestinal IELs of *C. perfringens*/*E. maxima* co-infected Ross chickens (NE) compared with uninfected controls (Control). Transcript levels were analyzed by microarray hybridization and qRT-PCR in both groups. Each bar represents the mean ± SD value of the fold change in the transcript level (n = 3). ANXA1, Annexin A1; APP, Amyloid precursor protein; ARHGEF6, Rho guanine nucleotide exchange factor 6; CALB1, Calbindin 1; COL1A2, Collagen, type 1, alpha 2; CXCL14, Chemokine (C-X-C motif) ligand 14; GJA1, Gap junction alpha 1; HSP90B1, Heat shock protein 90kDa beta 1; VEGFA, Vascular endothelial growth factor A; ALCAM, Activated leukocyte cell adhesion molecule; MTTP, Microsomal triglyceride transfer protein; LCP1, Lymphocyte cytosolic protein 1; TNFSF11B, Tumor necrosis factor superfamily 11; SERPINE1, Serpin peptidase inhibitor, clade F1.

The transcript expression patterns observed by microarray analysis between *C. perfringens*/*E. maxima* co-infected Ross chickens and uninfected controls were validated by qRT-PCR for 15 immune-related mRNAs. The levels of all transcripts were consistently increased or decreased by each method of quantification (Fig. 3). The some cases, differences in the magnitude of the changes observed by the two techniques might be related to differences in the normalization methods used and/or the different fluorescent dyes used [19].

### Body weight gain, oocyst shedding, and immune-related gene expression during experimental NE in ADOL line 6 and line 7 chickens

**Figure 4:**
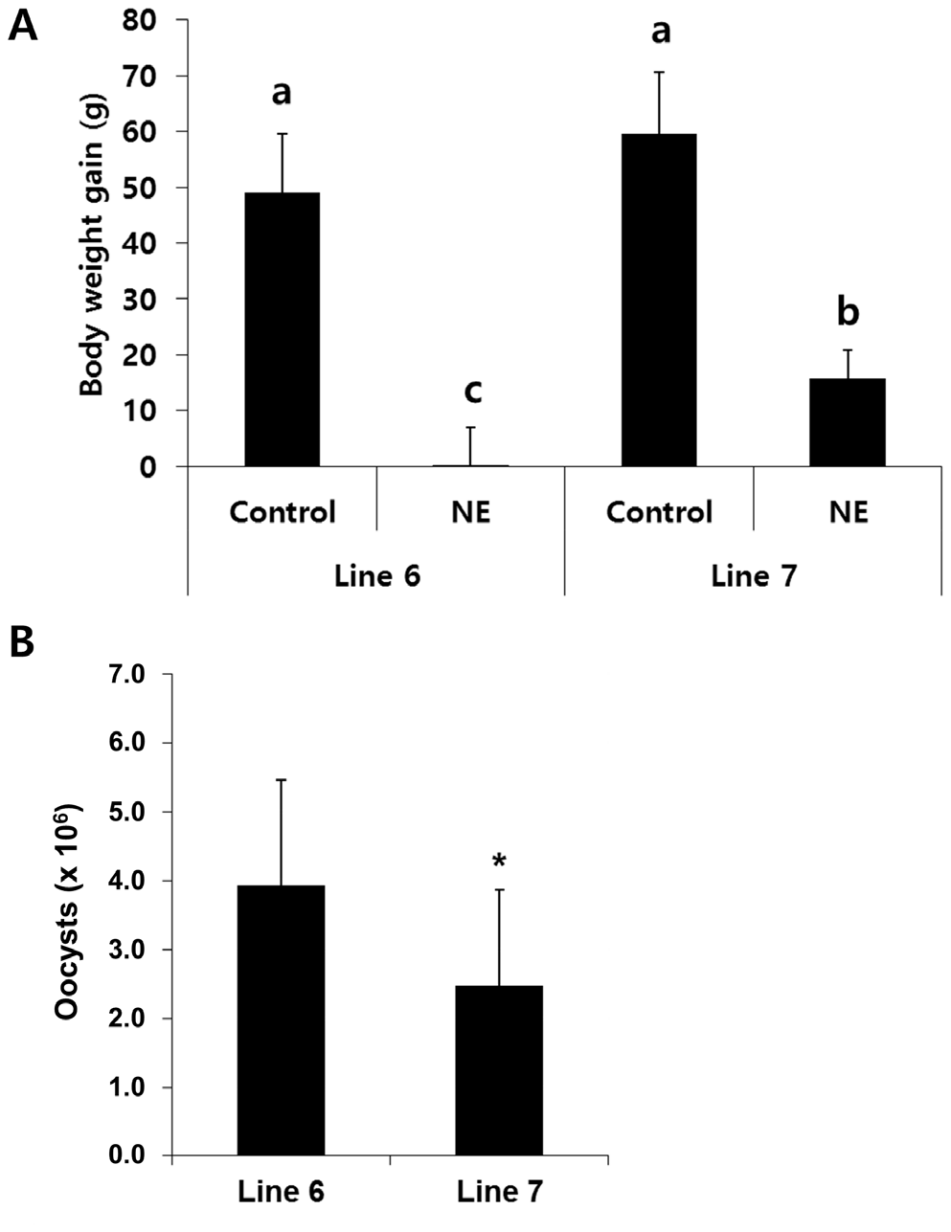
Effect of *C. perfringens*/*E. maxima* co-infection of ADOL line 6 and line 7 chickens on body weight gains and oocyst shedding. (A) Chickens were uninfected (Control) or co-infected with *C. perfringens* and *E. maxima* (NE) and body weight gains were measured between days 0–9 post-infection with *E. maxima*. Bars with different letters are significantly different according to the Duncan's multiple range test (*P*<0.05). (B) Fecal oocyst shedding was measured between days 0–9 post-infection with *E. maxima* in *C. perfringens*/*E. maxima* co-infected chickens. Each bar represents the mean ± SD value (n = 8). *, *P*<0.05.

Line 6 chickens had reduced body weight gains between days 0–9 post-infection with *E. maxima* (line 6 gain, 0.5±7.0 g; line 7 gain, 16±5.0 g) (Fig. 4A). Body weight gains between these same time points were equal in the two lines in uninfected birds. Co-infected line 6 chickens had greater fecal oocyst shedding between days 0–9 post-infection with *E. maxima* compared with line 7 chickens (line 6 oocyts, 3.9±1.5×10^6^; line 7 oocysts, 2.5±1.4×10^6^) (Fig. 4B). These results suggest that line 6 chickens have increased susceptibility for the development of experimental NE compared with line 7 chickens. To characterize the pattern of immune-related gene expression that might be related to this observed difference in disease susceptibility, the levels of transcripts for 21 selected genes were analyzed by qRT-PCR in uninfected and *C. perfringens*/*E. maxima* co-infected line 6 and line 7 chickens. Of these, 15 transcripts were expressed at greater levels in co-infected line 6 chickens compared with line 7 birds, 5 were equal in the two lines, and 1 was decreased in co-infected line 6 *vs*. line 7 chickens (Table 6).

**Table 6:** Immune-related intestinal IEL transcript levels in uninfected and in *C. perfringens*/*E. maxima* co-infected ADOL line 6 and line 7 chickens.

Gene	Treatment	ADOL Line 6	ADOL Line 7
ANXA1	Uninfected	3.6E-03±2.5E-04^c^	6.5E-03±4.5E-04^b^
	NE	1.0E-02±7.4E-05^a^	1.6E-03±9.4E-05b^c^
ARHGEF6	Uninfected	1.9E-03±7.8E-06^c^	2.4E-03±6.9E-05^b^
	NE	3.3E-03±1.0E-04^a^	2.6E-03±9.9E-05^b^
LITAF	Uninfected	2.0E+00±3.6E-01^b^	6.7E-01±2.6E-02^c^
	NE	4.8E+00±2.7E-01^a^	8.3E-01±3.7E-02^c^
SOCS3	Uninfected	1.3E-08±1.9E-09^b^	4.0E-07±1.6E-08^b^
	NE	3.0E-06±1.1E-07^a^	9.2E-07±3.5E-08^b^
BCL2	Uninfected	2.7E-04±1.6E-05^b^	5.1E-04±2.1E-05^a^
	NE	2.2E-04±7.4E-06^b^	1.3E-04±9.3E-06^c^
GJA1	Uninfected	5.5E-05±3.5E-06^a^	1.8E-05±3.4E-06^b^
	NE	1.2E-05±3.7E-07^b^	1.6E-05±9.7E-07^b^
IL8	Uninfected	1.5E+00±1.0E-01^a^	7.2E-01±6.3E-02^b^
	NE	5.7E-01±1.1E-02^b^	4.4E-01±6.2E-02^b^
SOCS6	Uninfected	7.5E-04±3.9E-05^a^	2.6E-04±6.7E-06^b^
	NE	2.6E-04±9.7E-06^b^	2.3E-04±8.1E-06^b^
HSP90B1	Uninfected	1.2E-02±2.1E-04^b^	1.3E-02±3.0E-04^b^
	NE	1.6E-02±3.8E-04^a^	3.9E-03±8.1E-05^c^
VEGFA	Uninfected	2.6E-02±3.6E-04^b^	2.8E-02±6.0E-04^b^
	NE	4.1E-02±4.0E-04^a^	1.6E-02±3.1E-04^c^
MTTP	Uninfected	6.1E-06±1.7E-07^bc^	3.1E-06±9.4E-08^c^
	NE	2.9E-05±6.2E-07^a^	9.4E-06±7.2E-07^b^
SERPINF1	Uninfected	3.8E-04±2.2E-05^b^	1.5E-03±2.7E-05^a^
	NE	7.7E-05±2.1E-06^c^	1.3E-04±8.0E-06^c^
CALB1	Uninfected	2.6E+00±7.5E-02^a^	7.3E-01±3.5E-02^b^
	NE	3.7E-03±6.0E-05^c^	5.7E-02±9.2E-03^c^
LCP1	Uninfected	3.4E-06±2.8E-07^c^	2.8E-06±3.5E-07^c^
	NE	3.2E-05±3.8E-07^a^	4.6E-05±3.9E-06^b^
TNFRSF11B	Uninfected	1.9E-07±9.5E-09^a^	1.3E-07±2.3E-08^b^
	NE	1.0E-07±9.2E-09^b^	3.3E-08±5.4E-09^c^
COL1A2	Uninfected	2.9E-04±2.8E-05^ab^	3.7E-04±2.6E-05^a^
	NE	1.2E-04±7.3E-06^c^	2.4E-04±2.7E-05^b^
TCF12	Uninfected	2.3E-04±1.4E-05^a^	1.1E-04±1.2E-05^b^
	NE	1.6E-04±2.7E-05^b^	6.1E-05±3.5E-06^c^
APP	Uninfected	1.9E-02±1.2E-04^a^	1.2E-02±3.0E-04^b^
	NE	1.1E-02±3.0E-04^b^	5.1E-03±1.4E-04^c^
CXCL14	Uninfected	9.6E-04±5.4E-05^a^	2.6E-04±1.5E-05^b^
	NE	3.1E-04±7.7E-05^b^	5.2E-05±4.8E-06^c^
NFKBIZ	Uninfected	2.4E-03±9.9E-05^b^	6.0E-03±1.6E-04^a^
	NE	1.8E-03±4.6E-05^c^	1.3E-03±1.5E-05^d^
TAB3	Uninfected	1.9E-03±1.2E-05^a^	1.2E-03±1.2E-05^b^
	NE	4.4E-04±1.6E-05^c^	2.4E-04±1.1E-05^d^
TRAF3	Uninfected	1.9E-03±3.4E-05^a^	6.8E-04±5.1E-06^b^
	NE	6.0E-04±1.9E-05^b^	2.5E-04±9.7E-06^c^

Each value represents the mean ± SD (n = 3). Within each gene group, values with different superscripts are significantly different according to the Duncan's multiple range test (*P*<0.05).

## Discussion

This study was conducted to investigate the host response to avian NE, and the role of host genetics in this response, using an experimental model of *C. perfringens*/*E. maxima* co-infection. Microarray hybridization identified 1,049 transcripts whose levels were altered in intestinal IELs of co-infected Ross chickens compared with uninfected controls, the majority of which (57.3%) were increased. From these differentially expressed genes, 5 biological functions, all related to host immunity, and 11 pathways were identified. ADOL line 6 chickens were more susceptible to NE compared with the MHC-identical line 7, as revealed by decreased body weight gain and increased *E. maxima* oocyst shedding. Of note, body weight gains of the experimentally inbred ADOL chickens are lower compared with those of Ross broilers which have been specifically bred for rapid, high efficiency growth rate. Of 21 pro- and anti-inflammatory genes examined, 15 were increased in line 6 *vs*. line 7 chickens. It is interesting to note that most of immune-related transcripts examined (71%) were increased in the more disease susceptible line 6 chickens.

Identification of antibiotic-free disease control strategies against NE has been hindered due, in large part, to the difficulty of experimentally reproducing the disease by *C. perfringens* infection alone [20], [21]. Nevertheless, several prior studies have profiled chicken transcriptome changes following infection by *C. perfringens* alone [22], [23]. Functional genomics using the *C. perfringens*/*E. maxima* co-infection method offers one possibility to investigate the host response to experimental disease, and to the best of our knowledge, this study is the first report of such an approach. We chose to analyze transcriptome alterations using intestinal IELs because they are the primary immune effector cells of the gut-associated lymphoid tissues which recognize and destroy pathogens that breach the intestinal epithelial barrier [24]. Chicken intestinal IELs are composed of two phenotypically and functionally distinct subpopulations, natural killer cells and T-lymphocytes [25], and both cell types are major effectors against *C. perfringens* and *Eimeria* spp. [26].

In addition to infection of the chicken gut, *C. perfringens* infection also can be manifested as gangrenous dermatitis, a necrotizing skin disease accompanied by severe cellulitis of the subcutaneous tissues [27]. In a previous study, we characterized gene expression profiles in chickens with gangrenous dermatitis and identified “inflammatory response” as the most significantly affected biological functions associated with this disease [27]. Therefore, it was not unexpected to find in the current study that the differentially regulated genes in intestinal IELs from chickens with experimental NE also were associated with biological functions and networks related to “inflammatory response” and “immune response”. Interestingly, all of the remaining significantly annotated biological functions in *C. perfringens*/*E. maxima* co-infected Ross birds were related to movement or quantity of leukocytes. Further, the most reliable canonical pathway identified was “leukocytes extravasation signaling”. Leukocyte extravasation from the vasculature into infected interstitial tissues constitutes a key component of the host inflammatory response. Inflammatory cytokines and proteases, particularly matrix metalloproteinases (MMPs), are released by extravasating cells to modify the extracellular matrix [28]–[30]. Our results now establish that both matrix MMP-7 and MMP-9 were up-regulated in intestinal IELs of co-infected birds compared with uninfected controls. Collectively, the results suggest that the movement and/or quantity of leukocytes regulate host resistance to experimental avian NE.

ADOL lines 6 and 7 are homozygous for the MHC *B^2^* haplotype, but differ in MDV and ALV resistance [31], [32]. Line 6 chickens are susceptible to both MDV and ALV infection, whereas line 7 chickens are relatively resistant to infection by these viruses. The current study now extends the differential response of these two lines to experimental NE, where line 6 chickens showed greater susceptibility to *C. perfringens*/*E. maxima* co-infection compared with line 7 birds. Of the 21 immune-related transcripts analyzed in line 6 and line 7 birds, 15 were increased in the more disease susceptible line 6 chickens. The levels of 9 of these up-regulated transcripts also were examined in Ross chickens, where 8 were increased following *C. perfringens*/*E. maxima* co-infection compared with uninfected controls (ANXA1, HSP90B1, VEGFA, MTTP, TNFSF11B, TCF12, APP, and CXCL14). In humans, annexin A1 (ANXA1) regulates tumor necrosis factor-α (TNF-α)-induced cell proliferative and inflammatory responses [33]. In other human studies, annexin A1 protein acted as a positive regulator of MMP-9 expression, as well as metastatic invasion of breast cancer cells, through activation of NF-κB signaling [34]. The expression of LPS-induced TNF-α factor (LITAF), a transcription factor that regulates inflammation through transcriptional activation of TNF-α [35], [36], also was increased in *C. perfringens*/*E. maxima* co-infected line 6 chickens compared with line 7 birds. Counteracting these proinflammatory effects, suppressors of cytokine signaling (SOCS) proteins act in an anti-inflammatory manner by providing a negative feedback loop to attenuate cytokine signaling. Mouse TNF-α, and by inference chicken LITAF, were responsible for the maintenance of SOCS3 expression *in vitro* and TNF-α deficiency in murine macrophages accelerated the degradation of SOCS3 protein [37]. In the current study, LITAF and SOCS3 were increased in co-infected ADOL line 6 chickens compared with line 7 birds. Therefore, the susceptibility to experimental avian NE might be determined, at least in part, by interactions among ANXA1, LITAF, SOCS, and/or other proteins encoded by genes that are differentially regulated in line 6 *vs*. line 7 chickens during experimental NE. Future studies are needed to provide additional insights into the host-pathogen interactions in avian NE that might facilitate the development of effective control strategies against field infections by clostridial bacteria.

## Supporting Information

S1 Table Significant networks from the differentially expressed transcripts (>2.0 fold changes, P<0.05) following NE infection compared with uninfected controls.
(XLSX)
